# Multicenter registry and test bed for extended outpatient hemodynamic monitoring: the hemodynamic frontiers in heart failure (HF^2^) initiative

**DOI:** 10.3389/fcvm.2023.1321415

**Published:** 2023-11-29

**Authors:** J. Thomas Heywood, Kartik Munshi, Timothy Jordan, Evan Muse, Marat Fudim, Andrew J. Sauer, Margaret McDermott, Hirak Shah, Arvind Bhimaraj, Rola Khedraki, Monique R. Robinson, Patrick McCann, Elizabeth Volz, Ashrith Guha, Orvar Jonsson, Kunjan A. Bhatt, Mosi K. Bennett, Terrie Ann Benjamin, Maya Guglin, Jacob Abraham

**Affiliations:** ^1^ Division of Cardiovascular Medicine, Scripps Clinic, Prebys Cardiovascular Institute, La Jolla, CA, United States; ^2^ Department of Cardiovascular Medicine, University of Kansas Medical Center, Kansas City, KS, United States; ^3^ Division of Cardiology, Duke Medical Center, Durham, NC, United States; ^4^ Saint Luke’s Mid America Heart Inst, Kansas City, MO, United States; ^5^ Houston Methodist DeBakey Heart and Vascular Center, Houston Methodist Hospital, Houston, TX, United States; ^6^ University Hospital Health System, Cleveland, OH, United States; ^7^ PRISMA Health, Columbia, SC, United States; ^8^ Division of Cardiology, UNC Rex Healthcare, Raleigh, NC, United States; ^9^ Sanford Cardiovascular Institute, Sioux Falls, SD, United States; ^10^ Department of Heart Failure, Austin Heart PA, Austin, TX, United States; ^11^ Allina Health Minneapolis Heart Institute, Minneapolis, MN, United States; ^12^ Heart Failure Division, M Health Fairview St. Joseph’s Hospital, St. Paul, MN, United States; ^13^ Indiana University, Indianapolis, IN, United States; ^14^ Center for Cardiovascular Analytics, Research, and Data Science (CARDS), Providence St. Joseph Research Network, Portland, OR, United States

**Keywords:** pulmonary artery pressure, pulmonary hypertension, heart failure, hemodynamics, remote monitoring, CardioMEMS

## Abstract

**Background:**

Hemodynamic Frontiers in Heart Failure (HF^2^) is a multicenter academic research consortium comprised of 14 US institutions with mature remote monitoring programs for ambulatory patients with heart failure (HF). The consortium developed a retrospective and prospective registry of patients implanted with a wireless pulmonary artery pressure (PAP) sensor.

**Goals/aims:**

HF^2^ registry collects demographic, clinical, laboratory, echocardiographic (ECHO), and hemodynamic data from patients with PAP sensors. The aims of HF^2^ are to advance understanding of HF and to accelerate development of novel diagnostic and therapeutic innovations.

**Methods:**

HF^2^ includes adult patients implanted with a PAP sensor as per FDA indications (New York Heart Association (NYHA) Class III HF functional class with a prior hospitalization, or patients with NYHA Class II or brain natriuretic peptide (BNP) elevation without hospitalization) at a HF^2^ member site between 1/1/19 to present. HF^2^ registry is maintained at University of Kansas Medical Center (KUMC). The registry was approved by the institutional review board (IRB) at all participating institutions with required data use agreements. Institutions report data into the electronic registry database using REDCap, housed at KUMC.

**Results:**

This initial data set includes 254 patients implanted from the start of 2019 until May 2023. At time of device implant, the cohort average age is 73 years old, 59.8% are male, 72% have NYHA Class III HF, 40% have left ventricular ejection fraction (LVEF) < 40%, 35% have LVEF > 50%, mean BNP is 560 pg/ml, mean N-Terminal pro-BNP (NTproBNP) is 5,490 pg/ml, mean creatinine is 1.65 mg/dl. Average baseline hemodynamics at device implant are right atrial pressure (RAP) of 11 mmHg, pulmonary artery systolic pressure (PASP) of 47 mmHg, pulmonary artery diastolic pressure (PADP) 21 mmHg, mean pulmonary artery pressure (mPAP) of 20 mmHg, pulmonary capillary wedge pressure (PCWP) of 19 mmHg, cardiac output (CO) of 5.3 L/min, and cardiac index (CI) of 2.5 L/min/m^2^.

**Conclusion:**

A real-world registry of patients implanted with a PAP sensor enables long-term evaluation of hemodynamic and clinic outcomes in highly-phenotyped ambulatory HF patients, and creates a unique opportunity to validate and test novel diagnostic and therapeutic approaches to HF.

## Introduction

Decompensated HF manifests through symptoms attributed to hemodynamic and neurohormonal abnormalities, leading to peripheral edema, pulmonary congestion, orthopnea, and paroxysmal nocturnal dyspnea (1). Zile et al. demonstrated that elevated filling pressures precede hospital admissions for HF and improve with therapy (2). Recent studies have shown that ambulatory PAP monitoring using the CardioMEMS^©^ system can reduce HF hospitalizations and improve quality of life by proactively managing hemodynamic changes compared to the conventional approach of reacting to signs or symptoms of HF (3–6).

Beyond its clinical utility, ambulatory hemodynamic monitoring offers unique platform for investigation into HF. While randomized trials of PAP sensors had follow-up for 6–18 months, patients often have these devices implanted for years. Given the paucity of longitudinal hemodynamic, treatment, and clinical outcomes in ambulatory patients with HF, the HF^2^ registry represents a deeply and uniquely phenotyped HF population (7). Moreover, this monitored population with a documented hemodynamic history (“hemodynamic transcript”) presents an opportunity for rapid and cost-effective testing of novel diagnostic and therapeutic approaches. In the early stages of drug development for renin-angiotensin-aldosterone system (RAS) blockers, invasive inpatient hemodynamic monitoring served as a proof of concept before large randomized controlled trials were conducted (8, 9). Although these trials were costly and involved a limited number of patients, they provided crucial data that informed the design and power calculations for subsequent pivotal outcome trials. In contrast, contemporary trials could be conducted faster, more cost-effectively, and with greater safety by assessing the effect of interventions on PAP, which represents a hemodynamic surrogate tied to improved clinical outcomes (10).

Against this backdrop, we have established a consortium of experienced CardioMEMS^©^ centers to create a comprehensive retrospective and prospective registry of patients implanted with a sensor. The HF^2^ registry capture extensive demographic, laboratory, ECHO and treatment information, hemodynamic data from the CardioMEMS^©^ device, and clinical events. The purpose of this manuscript is to provide a detailed description of the dataset and present information on the first 254 patients enrolled.

## Methods

### Study design and participants

The HF^2^ registry includes data from participating institutions on consecutive adult patients implanted with CardioMEMS^©^ as per FDA indications (NYHA Class III HF diagnosis with a prior hospitalization or patients with NYHA Class II or BNP elevation without hospitalization).

This registry was approved by the KUMC IRB (IRB study 00147383) and by local IRBs at participating institutions. KUMC and all participating institutions have signed an appropriate data use agreement. Participating institutions reported the data into the electronic registry database, which is built and maintained using REDCap data capture tools and housed at KUMC (11). Patient demographic data, blood test results, hemodynamic data from device implant, hemodynamic data from the CardioMEMS^©^, ECHO data, electrocardiogram (ECG) data, and clinical outcomes (HF hospitalizations, all-cause hospitalizations, emergency department (ED) visits, intravenous (IV) diuretic administration in the clinic, device-related complications, device recalibrations after initial implant, heart transplant, left ventricular assist device (LVAD), and death) are captured.

**Figure 1:**
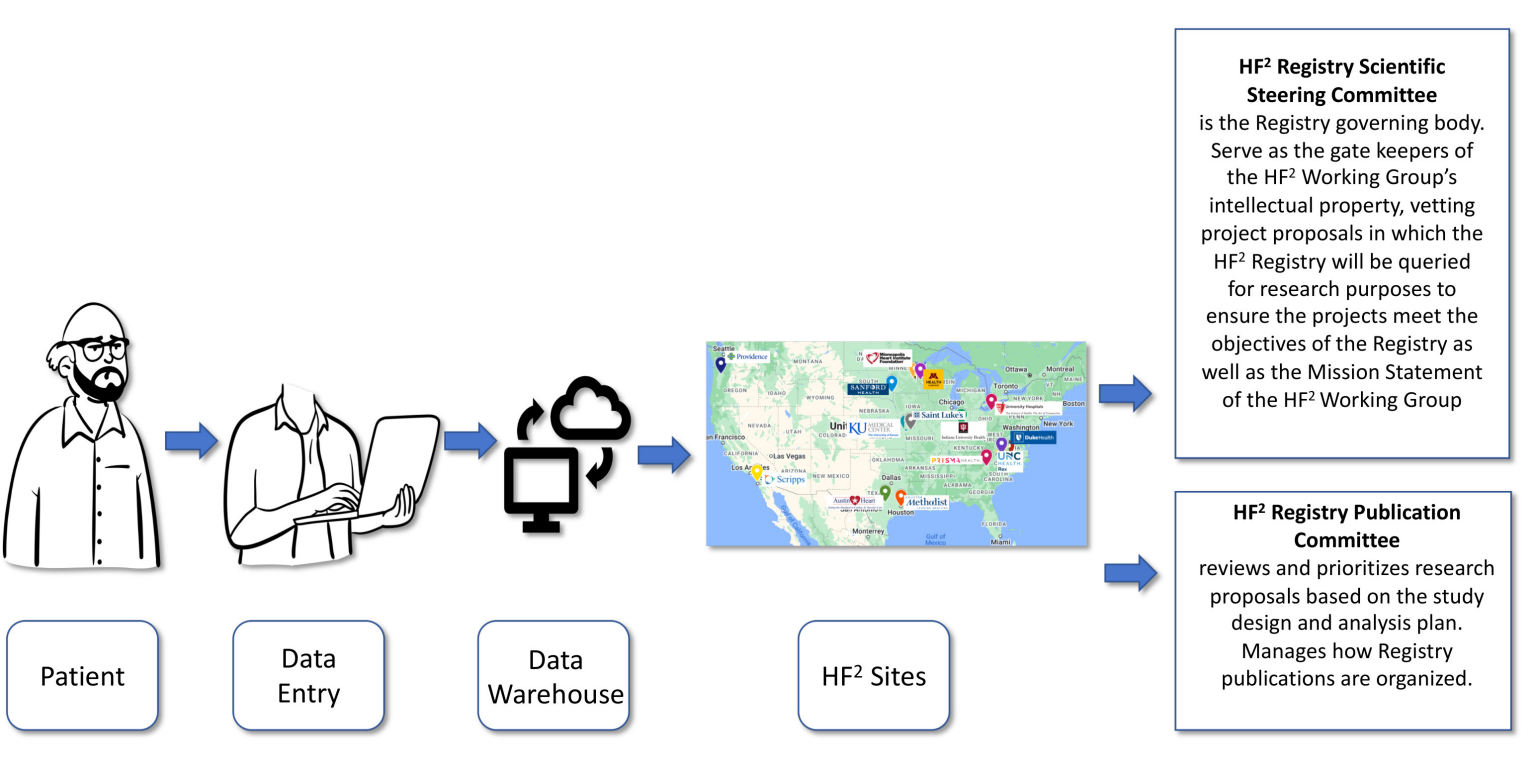
Workflow of the HF^2^ registry. The flow of data in the HF^2^ Registry from patient, to data entry, to the central Data warehouse, via individual institutions across the United States and overseen by both a scientific steering committee and a publication committee.

HF^2^ has a Registry scientific steering committee that is the governing body and serve as the gate keepers of the HF^2^ Working Group's intellectual property, vetting project proposals in which the HF^2^ Registry will be queried for research purposes to ensure the projects meet the objectives of the Registry as well as the Mission Statement of the HF^2^ Working Group. There is also a publication committee which reviews and prioritizes research proposals based on the study design and analysis plan and subsequently manages how Registry publications are organized (see Figure 1).

### Statistical analysis

Statistical analysis was done using IBM SPSS software version 29.0. Descriptive statistics were used to calculate frequencies, mean & median for the data variables used which include age, race, gender, comorbidity conditions, laboratory values, ECHO, NYHA class, implanted devices, medications and right heart catheterization data.

## Results

This initial report includes 254 patients implanted from the beginning of 2019 until May 2023 at 12 participating sites across the United States. See Table 1 for baseline demographic, comorbidity, laboratory, echocardiographic, and medication data. See Table 2 for initial hemodynamic measurements at the time of implant.

**Table 1:** Characteristics at implant.

Mean age	73
Male	59.8%
Female	40.2%
White	87%
African American	8%
Asian	3%
Other ethnicity	2%
CAD	59.4%
CKD	65%
Mean creatinine (mg/dl)	1.65 (range: 0.6–5.5)
Mean/median GFR (ml/min/1.73 m^2^)	Mean: 43.5, Median 41
Mean/median BMI (kg/m^2^)	Mean: 32, Median: 30.9
COPD	16.9%
Type II diabetes	46.9%
Atrial fibrillation	
Paroxysmal	38.2%
Permanent	16.9%
Persistent	7.9%
NYHA Class	
I	0.4%
II	22.8%
III	71.7%
IV	4.3%
Mean BNP (pg/ml)	560 (*n*: 36, range: 18–3,413)
Mean NTproBNP (pg/ml)	5,490 (*n*: 121, range: 20–45,200)
Left ventricular ejection fraction (LVEF)	
<40%	40.2%
41–50%	13.8%
>50%	34.6%
Missing LVEF at time of implant	11.4%
Device	
CRT-D	23.6%
CRT-P	3.5%
ICD	13.8%
Pacemaker	12.6%
No Device	46.5%
Medication	%Taking
ACE/ARB	20.1%
Aldosterone antagonist	53.5%
ARNI	29.9%
Beta blockers	74.4%
Inotrope	1.6%
Hydralazine	13.4%
Loop diuretics	96.9%
SGLT2i	24.4%
Thiazide	7.5%
As needed thiazide	13.4%
Nitrates	10.6%

Extensive baseline data at time of implant were recorded of these initial 254 patients and these are the highlights that include important demographic, comorbidities, NYHA Class, Laboratory, echocardiographic, device and medical therapies. BNP and NTproBNP use was institution dependent, see above corresponding patient numbers for each value.

CAD, coronary artery disease; CKD, chronic kidney disease; GFR, glomerular filtration rate; BMI, body mass index; COPD, chronic obstructive pulmonary disease; NYHA, New York heart association; BNP, brain-natriuretic peptide; NTproBNP, N-terminal pro-BNP; CRT-D, cardiac resynchronization therapy defibrillator; CRT-P, cardiac resynchronization therapy pacemaker; ICD, implantable cardioverter-defibrillator; ACE, angiotensin-converting enzyme inhibitors; ARB, angiotensin receptor blockers; ARNI, angiotensin receptor/neprilysin inhibitor; SGLT2i, sodium-glucose cotransporter-2 inhibitors.

**Table 2:** Hemodynamics at implant.

Hemodynamic values (units)	Mean
Right atrial pressure (mmHg)	11
Pulmonary artery systolic pressure (mmHg)	47
Pulmonary artery diastolic pressure (mmHg)	21
Mean pulmonary artery pressure (mmHg)	20
Pulmonary capillary wedge pressure (mmHg)	19
Cardiac output (L/min)	5.3
Cardiac index (L/min/m^2^)	2.6
Systemic vascular resistance (s/cm^5^)	1,794
Pulmonary vascular resistance (Wood unit)	2.5
Transpulmonary gradient (mmHg)	11
Pulmonary artery compliance (mL/mmHg)	3.2
Pulmonary artery pulsatility index	3.3
Mean arterial pressure (mmHg)	89
Cardiac power output (Watts)	1.1

Baseline hemodynamics that were recorded at time of initial pulmonary artery pressure sensor implant. Pulmonary artery compliance, defined by (Cardiac output/heart rate)/(Pulmonary artery systolic pressure—pulmonary artery diastolic pressure) X 1,000. Pulmonary artery pulsastility index, defined by (Pulmonary artery systolic pressure—Pulmonary artery diastolic pressure)/Right atrial pressure.

The mean age of patients is 73 years old, and 40.2% are female. Most patients are White (87%), with 8% African American, 3% Asian, and 2% other ethnicity. There is a high prevalence of coronary artery disease (CAD) (59.4%), atrial fibrillation (AF) (63%), chronic kidney disease (CKD) (65%), Chronic Obstructive Pulmonary Disease (COPD) (16.9%), and diabetes mellitus type II (DMII) (46.9%).

Most patients have NYHA Class III symptoms (71.7%), and the remaining have predominantly NYHA II symptoms (22.8%). Multiple lab parameters, including a complete metabolic panel, natriuretic peptides, hemoglobin, and hemoglobin A1c were recorded into the database. The cohort, on average has elevated BNP and NTproBNP levels, 560 pg/ml and 5,490 pg/ml, respectively. At time of implant, 40.2% of patients had an LVEF <40%, 13.8% of patients had an LVEF of 41%–50%, 34.6% of patients have an LVEF of >50%. Regarding device therapy, 23.6% of patients have a Cardiac Resynchronization Therapy Defibrillator (CRT-D), 3.5% have a Cardiac Resynchronization Therapy Pacemaker (CRT-P) device, 13.8% have an implantable cardioverter-defibrillator (ICD), and 46.5% have no device. 20.1% of patients are on angiotensin-converting enzyme (ACE) inhibitors or angiotensin receptor blockers (ARBs), 29.9% of patients are on angiotensin receptor/neprilysin inhibitor (ARNI), 74.4% of patients are on beta blockers (BB), 53.5% of patients are on aldosterone antagonists, 24.4% of patients are on sodium-glucose cotransporter-2 inhibitors (SGLT2i), 96.9% of patients are on some form of loop diuretic and 1.6% of patients are on inotrope therapy.

Mean hemodynamic values at the time of implant are listed in Table 2. Right-sided pressures were elevated with a mean RAP of 11 mmHg, there was mildly elevated left-sided pressures with a mean PCWP of 19 mmHg, and elevated pulmonary pressures with PASP/PADP of 47/21 mmHg and mPAP of 30 mmHg. CO and CI by indirect Fick were normal, 5.3 L/min and 2.6 L/min/m^2^, respectively. Systemic vascular resistance (SVR) mean was 1,794 s/cm^5^, pulmonary vascular resistance (PVR) mean was 2.5 Wood unit with a mean transpulmonary gradient (TPG) of 11mmHg. Pulmonary artery compliance, defined as (CO/heart rate)/(PASP—PADP) X 1,000, mean was 3.2 ml/mmHg. The mean pulmonary artery pulsastility index, defined as (PASP—PADP)/RAP, was 3.3.

## Discussion

**Figure 2:**
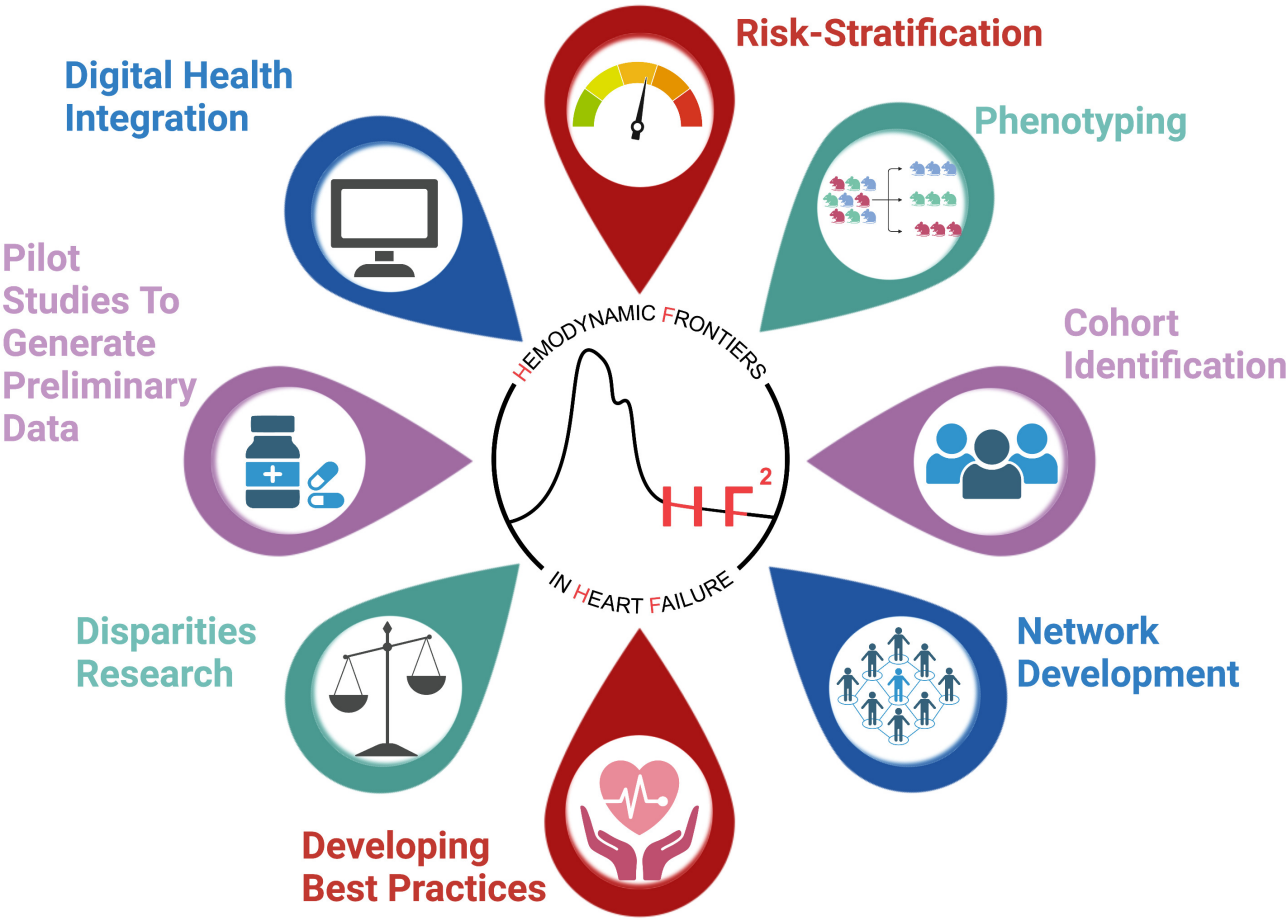
Central illustration demonstrating the utility of the HF^2^ registry as a test bed for multiple applications.

HF patients present with a wide array of comorbidities, devices, and ECHO and hemodynamic profiles and are treated with an increasingly complex therapeutic regimen. Through this registry of patients with the CardioMEMS^©^ device, our aim is to better understand this population of patients through the collection of relevant patient-level demographic, clinical, laboratory, echocardiographic and hemodynamic data. The creation of this registry will help facilitate future HF research by providing a platform to answer a variety of questions and test various therapeutic interventions (see Figure 2, Central Illustration).

In many ways, the 254 patients included in this report reflect real world HF patients that are often excluded from clinical trials. Our patient population is comparable to those in major preceding CardioMEMS© trials as well as recent pivotal HF trials, though with some notable differences. Our patients are older than patients enrolled in contemporary device HF trials (3–5, 12, 13). With 40% women, our cohort is more balanced for gender than the above trials, though similar to the EMPEROR trial (45% women) (14). In our cohort patients were predominantly White, 87%, though similar to the heart failure with preserved ejection fraction (HFpEF) group of GUIDE-HF which had 89% white patients (4, 13). The cohort predominantly includes patients with NYHA class III symptoms, with elevated baseline BNP or NTproBNP levels, as is comparable to GUIDE-HF, and suggests advanced disease and a high event rate (4, 13). There are almost equal portions of patients with HFpEF and a smaller portion of patients with mid-range ejection fraction (41–50%), also comparable to GUIDE-HF (4, 13). Our patients frequently have CAD, CKD, DMII, and AF, as is typical in hospitalized HF patients. However, 63% of our cohort have AF, which is higher than the aforementioned trials (3–5, 12–14). Also, our patients more frequently have CKD and lower GFR, which is more typical of real word HF patients. Just over half of patients had some type of device, either ICD or CRT. About 40% of patients had heart failure with reduced ejection fraction (HFrEF), and a large portion of patients were on appropriate guideline-directed medical therapy (GDMT) with ACE/ARB, ARNI, aldosterone antagonists, BB. Although only 24.4% of patients were on SGLT2i, the time of implant for a large portion of patients was prior to practice changing trials using these medications. Nearly the entire cohort, 97% of patients, were on loop diuretics of varying types, either furosemide, torsemide, or bumetanide. Baseline hemodynamic profiles at the time of implant further exemplify the heterogenous nature of HF patients. Patients, on average, had elevated RAP (11 mmHg) and PCWP (19 mmHg), with a mixture of pre, post, and mixed pulmonary hypertension, though patients did on average have normal CO. In our cohort, PASP was 47 mmHG, PADP was 21 mmHg and mPAP was 30 mmHg, similar to GUIDE-HF, with 45 mmHg, 19 mmHg, and 29 mmHg respectively (4, 13).

Having coalesced easily accessible and extensive data in our registry on this population of patients with the CardioMEMS© device, we will be able to better understand this group of patients through both retrospective research studies on the existing data and by collecting prospective data for future research studies. The registry formed by the ADHERE-HF group demonstrated the importance of following patients over time, and they were able to provide insights into the clinical characteristics, patterns of care, and outcomes of HF patients, while subsequently developing tools for the improvement of quality of care for HF patients (15).

Prior work by Lam et al, demonstrated the importance of pulmonary hypertension (PH) in patients with HFpEF (16). One of the first population-based estimates showed that PH was highly prevalent, often severe in HFpEF patients, and an adverse prognostic factor in HFpEF, independent of age (16). In addition, they proved that there was a pre-capillary component to these patients with HFpEF and PH, outside of the pulmonary venous congestion alone, suggesting a potential role in the treatment of pulmonary artery hypertension in the treatment of HFpEF (16). The majority of the registry patients have PH which could provide a convenient way to assess the reversibility of this process in left heart disease.

An extremely important goal of the registry will be to serve as a test bed for early HF therapies. Notably, two such cohorts with sacubitril/valsartan, and one with dapagliflozin, have taken these guideline directed medications and shown the ability to effectively decrease pulmonary pressures after a period of 4–6 weeks (10, 17, 18). As a consortium of HF cardiologists around the country, we will be able to combine ideas and hypothesis and rapidly test them utilizing the registry (6, 19, 20). With a test bed of HF patients, we can also enroll them in prospective studies to test a variety of drugs, approved or unapproved, and monitor their responses outside of large retrospective clinical trials. We can generate fast crossover trials to evaluate hemodynamic and cardiorenal effects of new therapies and compounds in HF that provide important data in early drug and device development in a period of months rather than years. This should speed product development and thus reduce costs.

### Limitations

The cohort of patients that remain in the database are those that are still alive, so future prospective research on this group of patients may be skewed by survivor bias. Patients with advanced CKD are limited in this data set given their inability to respond to diuretics and thus lower likelihood to be implanted with the CardioMEMS^©^ device.

## Conclusions

Presented here is the first cohort of HF patients implanted with the CardioMEMS© device in our multisite registry. A real-world registry of patients with extended hemodynamic monitoring can evaluate long-term outcomes in HF patients, provide data in unique patient groups, and provide an opportunity to evaluate diagnostic and therapeutic HF approaches in rapid turnaround cross over trials.

## Data Availability

The original contributions presented in the study are included in the article/Supplementary Material, further inquiries can be directed to the corresponding author.
